# Who Said That? The Effect of Hearing Ability on Following Sequential Utterances From Varying Talkers in Noise

**DOI:** 10.1177/23312165251320794

**Published:** 2025-02-24

**Authors:** Alexina Whitley, Timothy Beechey, Lauren V. Hadley

**Affiliations:** 1 Hearing Sciences - Scottish Section, 6123 University of Nottingham, Glasgow, UK

**Keywords:** hearing loss, multitalker listening, speech recognition, attention

## Abstract

Many of our conversations occur in nonideal situations, from the hum of a car to the babble of a cocktail party. Additionally, in conversation, listeners are often required to switch their attention between multiple talkers, which places demands on both auditory and cognitive processes. Speech understanding in such situations appears to be particularly demanding for older adults with hearing impairment. This study examined the effects of age and hearing ability on performance in an online speech recall task. Two target sentences, spoken by the same talker or different talkers, were presented one after the other, analogous to a conversational turn switch. The first target sentence was presented in quiet, and the second target sentence was presented alongside either a noise masker (steady-state speech-shaped noise) or a speech masker (another nontarget sentence). Relative to when the target talker remained the same between sentences, listeners were less accurate at recalling information in the second target sentence when the target talker changed, particularly when the target talker for sentence one became the masker for sentence two. Listeners with poorer speech-in-noise reception thresholds were less accurate in both noise- and speech-masked trials and made more masker confusions in speech-masked trials. Furthermore, an interaction revealed that listeners with poorer speech reception thresholds had particular difficulty when the target talker remained the same. Our study replicates previous research regarding the costs of switching nonspatial attention, extending these findings to older adults with a range of hearing abilities.

## Introduction

In everyday life, spoken conversation often takes place in noisy environments, from a rumbling car to a busy café. Listeners therefore need to selectively attend to, and comprehend, a specific talker of interest while ignoring the background noise. This can be particularly challenging when said background noise includes speech (i.e., “cocktail party listening”) which masks the target speech and requires competing linguistic information to be inhibited (Conway et al., 2001) Further complicating the task is the fact that in conversation, the talker of interest can change, and hence inhibition of other voices may need to be reduced around turn switches. Indeed, sequential utterances from a single talker have been shown to be comprehended better than sequential utterances spoken by multiple talkers (Bressler et al., 2014; Choi & Perrachione, 2019; Lim et al., 2019), indicating the impact of conversational turn taking processes on speech understanding. Hence to comprehensively understand conversational listening in daily life, it is important to investigate how listeners understand sequential utterances, spoken by different talkers, particularly in nonideal listening conditions. The papers that have addressed how listeners follow sequential utterances typically investigate young, normal hearing, adults (Lin & Carlile, 2015; 2019). However, as age and hearing loss may affect the ability to segregate competing speech (Lee & Humes, 2012; Mackersie et al., 2011), we extend this prior work to investigate whether the commonly reported communication difficulties in social settings reported in these populations may be driven by speech processing issues specific to such conversation-type listening processes. We therefore used a multiutterance speech listening task, in which we varied whether the target talker remained the same or changed, to assess the effect of hearing and age on listeners’ ability to follow sequential utterances in the presence of noise.

### Conversation at a Cocktail Party

Cocktail party listening poses a challenge for both auditory and cognitive processes. In such a situation, the listener must first segregate sound sources into distinct auditory streams using distinguishing features such as spatial separation and voice characteristics (Allen et al., 2008), and then use these cues to focus their attention on the stream of interest (and inhibit the competing stream). Although it is common for interlocutors to vary in group conversation, a variety of work has shown that having a consistent target talker enhances the speech intelligibility in multitalker backgrounds (Brungart & Simpson, 2007; Lin & Carlile, 2019). Such consistency may allow listeners to better model a talker's vocal characteristics, with prior exposure readying their auditory system for efficient streaming and comprehension of the target (Samson & Johnsrude, 2016). Indeed, using the coordinate response measure (CRM), Brungart and Simpson (2007) found improved recall when the target voice remained the same between trials compared to when it changed. Furthermore, knowing where to expect the target voice also contributes to speech intelligibility, with a consistent talker location also benefitting the listener (Lin & Carlile, 2015).

Yet in conversation, “dynamic” situations are common, with the talker varying moment-to-moment. In this case, the tuning of one's auditory system to the voice heard immediately earlier does not help: when the talker changes streaming is disrupted and the listener must rapidly adapt to the new target's vocal characteristics. Thus, listening to varying talkers involves additional cognitive resources, both due to the need to monitor for potential targets, and to switch attention once the appropriate target is identified. Several studies have shown that following such changing talkers comes at a perceptual “cost,” with talker voice, talker location, and probability of switching each contributing to the challenge (Best et al., 2008; Brungart & Simpson, 2007; Lin & Carlile, 2015). These costs have been proposed to relate to the combination of disengaging attention from a prior talker/location, and reengaging attention to the new talker/location (Lin & Carlile, 2015; 2019).

One particularly salient study investigating the effect of talker switches was conducted by Lin and Carlile in 2019. This study presented two target sentences sequentially, identifiable by the use of a common name (e.g., “Nina gives two big shoes” and “Nina has six old toys”). The first target sentence was presented in silence, followed by a 300 ms gap, after which a second target sentence occurred in the presence of an overlapping masking sentence (offset by ±100 ms). The voice could remain the same between target sentences (same voice), could change (new voice), or could change while additionally retaining the original target voice as the masker (old voice). Stimuli were presented diotically over headphones removing the influence of spatial cues, and recall of targets was measured via yes/no responses to probe words from the sentences. Young normal hearing listeners experienced a significant cost of the target talker switching, whereby recall for the second target sentence was significantly better when the voice remained constant than when it switched. Furthermore, they showed significantly more masker confusions when the original target became the masker than when the target remained constant, suggesting additional difficulty which could be related to disengaging from a prior target.

While this paradigm is highly constrained to allow analyses of subtle differences in performance, it nonetheless captures many of the processes involved in conversation. Each trial is analogous to following a conversational turn switch, with the gap between utterances being of a typical duration in conversation (cf., Stivers et al., 2009), and thus allows a nuanced analysis of selective attention to sequential target sentences based on linguistic cues (i.e., the name mentioned by the talker). While it may not be common to hear two talkers starting after a gap at approximately the same time in an otherwise quiet environment (without one of them yielding the floor to the other), presenting the second sentence in a competing speech masker reduces ceiling effects of recall and allows analysis of attention to the correct target as well as confusions with the masker. Analyzing both the accuracy of listeners’ recall and the types of errors that they make provides a valuable means of addressing the processes involved in following conversations that cannot be tapped by isolated-sentence paradigms.

### Effects of Hearing Loss

In this article, we are particularly interested in the effects of aging and hearing loss on a listener's ability to follow sequential utterances. Older hearing-impaired listeners may have increased difficulty following conversations in complex multitalker environments both due to reduced audibility, and also to deficits in monitoring and ignoring competing streams (Getzmann et al., 2015; Meister et al., 2020; Oberem et al., 2017; Wächtler et al., 2022). While age-related changes in peripheral hearing may lead to increased difficulty separating target speech from competing talkers, there are additional reasons to anticipate that hearing loss would specifically impact conversation following in cocktail party situations.

Individuals with hearing impairment may differ in their ability to take advantage of consistent voice characteristics to selectively attend to speech. Reduced spectro-temporal resolution could disrupt the ability to discriminate between target and masker, with previous studies showing that hearing loss reduced the ability to use pitch and vocal tract cues (formant frequencies) to segregate competing sentences (Mackersie et al., 2011; Summers & Leek, 1998). As such, listeners with hearing loss may have greater difficulty forming distinct auditory objects and selectively attending to specific sources of interest (Shinn-Cunningham & Best, 2008). Such difficulty selectively attending to target speech would be particularly detrimental during conversation, and indeed older adults with hearing loss report finding it particularly difficult to follow conversational turns occurring after a switch in talker (based on an analysis of >1,000 responses collected in our lab to the SSQ question “You are with a group and the conversation switches from one person to another. Can you easily follow the conversation without missing the start of what each new speaker is saying?”). In conversation, reduced auditory object formation is likely to cause listeners to miss portions of the desired speech stream and struggle to rapidly switch between talkers (Shinn-Cunningham & Best, 2008).

Furthermore, hearing loss has also been related to increased listening effort, which may reduce available resources during listening and further impact speech processing (Pichora-Fuller et al., 2016); Rönnberg et al., 2013). For example, a study by Meister and colleagues showed that while listeners with hearing loss had relatively accurate speech recognition when attending to a single talker in the presence of a competitor, they showed poorer recall accuracy and increased substitution errors when trying to attend to both talkers simultaneously compared to listeners with normal hearing (Meister et al., 2016). This could reflect increased load due to sensory impairment interfering with memory processes during the shared attention task, but not audibility during selective attention. Thus, not only audibility, but also the cognitive mechanisms involved in attending to speech may be impacted by hearing loss.

A key study by Wächtler and colleagues began to address the effects of age and hearing when listening to utterances switching between talkers. In this work, target sentences were always indicated by the name “Stefan,” were each masked by two other concurrent talkers, and were presented one by one (Wächtler et al., 2022). Participants repeated each target sentence back once it ended, and then the next target occurred. They compared errors between a “static” condition, where the target talker remained the same, and a “dynamic” condition, where the target talker switched between trials 20% of the time. In the “dynamic” condition, recall accuracy (namely error rates) for target sentences was analyzed in relation to whether the target talker in the prior trial was the same (nonswitch trial) or was new (switch trial). Participants were grouped into young normal hearing, old normal hearing, or old with a mild–moderate hearing loss. While all listeners made more errors after a talker switch, it was only when the talker remained the same that there was an interaction with hearing loss: hearing loss impaired recall in trials directly before a talker switch (in relation to the young normal hearing group) but did not show any effect in trials after a talker switch. This extends the work of Meister et al. (2020) to suggest that the load associated with monitoring multiple sources is especially pronounced in hearing-impaired listeners. However, this study only saw differences between the older hearing-impaired group and the young normal hearing group, making it difficult to disentangle the effects of age and hearing ability. In addition, following a switch, the proportion of errors originating from the old target talker versus the other nontarget talker did not significantly differ—suggesting listeners had no difficulty disengaging from the previous target talker contrary to the findings by Lin and Carlile (2019; and also Brungart & Simpson, 2007). However, Wächtler's paradigm required recall of target sentences presented one-by-one, inserting breaks between utterances, which may have increased the difficulty of using a talker's voice beneficially due to greater reliance on memory resources. As the study by Lin and Carlile (2019) required recall of two sentences presented in quick succession before and after a turn-taking gap, their paradigm may be more reflective of typical conversational speech processing.

### Motivation for the Current Study

The present study brings together the paired turn following paradigm of Lin and Carlile (2019) and the investigation of hearing loss effects begun by Wächtler et al. (2022). We extend the prior work on hearing loss by investigating both the reengagement and disengagement of attention (through the use of Lin and Carlile's same, new, and old talker conditions), and using a continuous measure of hearing ability (i.e., speech in noise performance). Critically, the present study focuses on the recall of two sequential sentences interspersed by a short gap to better approximate conversational turn following and includes steady-state noise conditions to assess whether poorer performance with hearing loss can be explained as a simple audibility issue.

As described above, we use a turn-following paradigm that is in several ways analogous to following everyday conversational turn taking: sequential utterances are separated by a gap, the talker of each utterance can vary, specific content is consistent between utterances (the name), and interfering noise can be present. We conducted this study online with young normal hearing adults to ensure replication of prior work (Lin & Carlile, 2019), as well as with older adults with a range of hearing abilities to address the effect of hearing loss. We replicate previous studies reporting the effects of talker switches and hearing ability on speech recall accuracy, and critically demonstrate that listeners with worse hearing are particularly poor at recalling target words when the target talker remains the same.

## Methods

### Participants

Participants were recruited via Prolific (www.prolific.com) to an online survey containing questions about their hearing and a hearing test. We ran this study as a two-session experiment, the former including some hearing questionnaire assessments to screen appropriate participants for the main turn-following task (detailed in Procedure). We present data from a total of 22 young adults with self-reported “good” hearing (10 male, 12 female, *M*_age_ = 26.38), and 43 older adults with a range of self-reported hearing abilities (27 male, 16 female, *M*_age_ = 70.40). Of the 43 older adults, 22 were categorized as “good” hearing (13 male, 9 female, *M*_age_ = 70.18), and 21 as “poor” hearing (13 male, 8 female, *M*_age_ = 70.62).

While a further 62 participants were originally recruited to session 1, 35 did not pass our screening criteria, 19 declined to continue, 3 provided incomplete data in the experimental task, and 5 were removed as outliers in the experimental task (detailed in Procedure).

Ethical approval was obtained from the University of Nottingham Faculty of Medicine and Health Science Research Ethics Committee (REC reference: FMHS 423-1221). Participants provided written informed consent to participate in this study.

### Measures of Hearing Abilities (Session 1)

Table 1.

**Table 1.** Description of Hearing Measures.

Measure name	Description
Digit triplet test (DTT; (Smits et al., 2004)	Spoken numbers presented in a background of steady-state speech-shaped noise using an adaptive track procedure (25 trials, starting at an SNR of −16 dB, adjustments in 2 dB steps). Speech-reception threshold (SRT) calculated as an average of the SNR in the last 19 trials. Lower SRTs indicate better hearing in noise thresholds.
Speech, spatial, and qualities of hearing scale-12 (SSQ-12; Noble et al., 2013)	Self-report measure of hearing abilities in everyday life. Higher scores indicate better perceived hearing abilities.
Hearing Handicap Inventory for the Elderly (HHIE; Ventry & Weinstein, 1982)	Self-report measure designed to assess the emotional and social effects of hearing loss for older adults. Higher scores indicate greater presence of perceived emotional and situational hearing handicaps. Scores ≥17 indicate mild to moderate handicap and ≥43 indicate a significant handicap.

### Turn-Following Task (Session 2)

#### Stimuli

**Figure 1.**
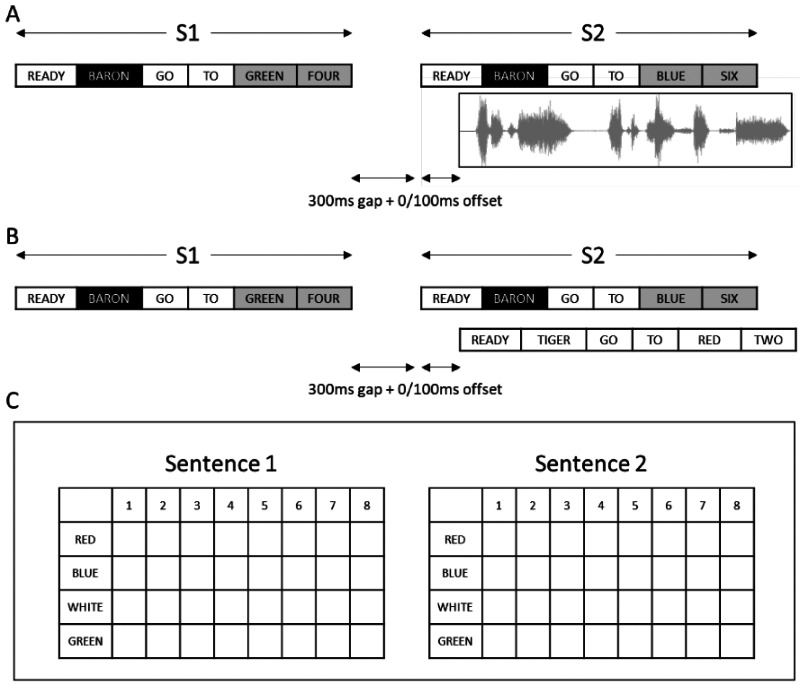
(A) Example of trial with a noise masker. (B) Example of trial with a speech masker. Target sentences (S1 and S2) are assigned an identical callsign. (C) Response screen that followed each trial.

Speech stimuli included sentences from the CRM corpus (Bolia et al., 2000). CRM sentences are comprised of interchangeable callsigns (i.e., names), colors, and numbers, taking the form “Ready [callsign] go to [color] [number] now.” There were eight two-syllable callsigns, four single-syllable colors (blue, red, green, white), and seven single-syllable numbers (1, 2, 3, 4, 5, 6, and 8). All sentences were spoken by three female talkers. Each trial consisted of two target sentences presented one after the other, each with the same callsign. The first sentence was presented in quiet, and the second sentence was presented concurrently either with a masking noise (Figure 1A), or a competing masker sentence (Figure 1B) at the same RMS level as the target sentence (i.e., 0 dB SNR). Participants completed 140 trials in total (details below).

For conditions involving noise masking, we applied amplitude modulation based on the CRM sentences to speech-weighted noise. The speech-weighted noise used the long-term spectrum generated from the speech data of Byrne et al. (1994). Using MATLAB, the envelope of the CRM sentences was then extracted and applied to the speech-weighted noise. Speech envelopes were generated from each talker for each combination of masker, color, and number. Therefore, the modulated speech-weighted noise has a similar average long-term spectrum, as well as equivalent temporal fluctuations in the CRM sentences, providing a condition with similar energetic masking without including intelligible speech information. Noise samples were randomized for each trial. For conditions involving speech masking, masking sentences were generated from the CRM corpus and randomized for each trial.

As in Lin and Carlile (2019), a 300 ms gap was included between the end of the first sentence and the start of the next stimulus (either the target or the masker) to replicate approximate turn taking duration of English speech and thus provide realistic task demands relevant to conversation (Stivers et al., 2009). The second target sentence and the masker were offset from each other by 100 ms (i.e., one began 300 ms after sentence 1, and one began 400 ms after sentence 1), counterbalanced such that half the trials involved the target sentence beginning first, and half involved the masker sentence beginning first.

Following each trial participants were presented with a response screen (Figure 1C). The response screen contained two grids; one for sentence 1 and one for sentence 2. Participants were required to select the corresponding color–number coordinate heard in both target sentences. Participants did not receive feedback on whether they had selected the correct words.

#### Experimental Conditions

**Figure 2.**
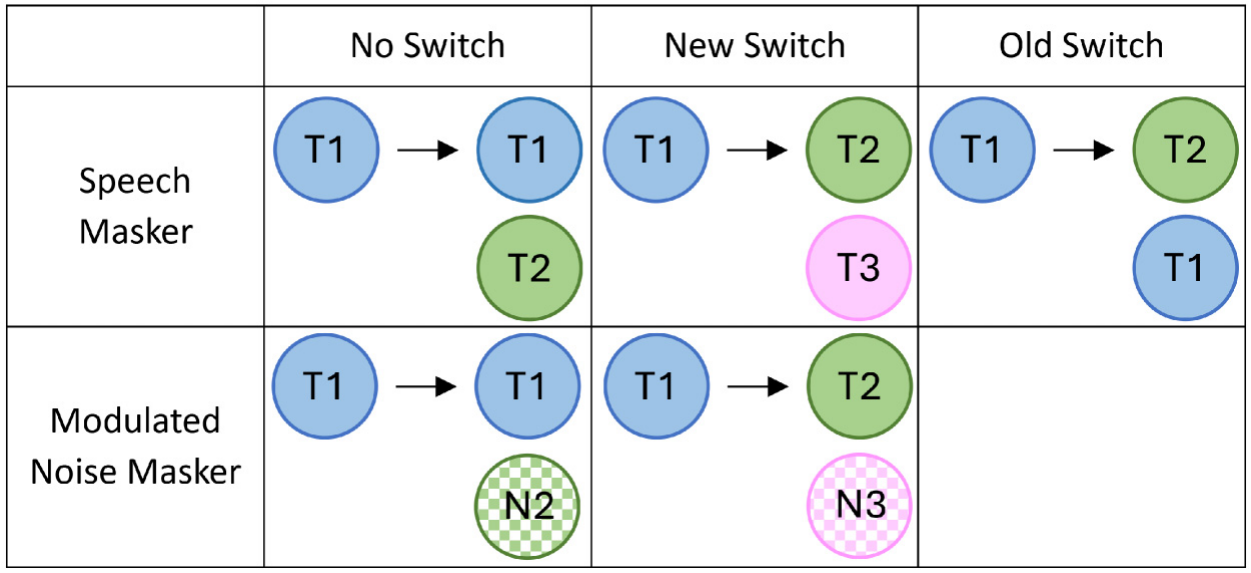
Experimental conditions. *Note*: Example of talker combinations for target and masker sentences. Talkers (3 Females) are denoted by different colors (T = talker, N = speech modulated noise).

We included the three speech masking conditions used in Lin and Carlile (2019), as well as two additional noise masking conditions (Figure 2). In the speech masking conditions, the target talker of the two sentences either (1) remained the same, with a different talker masker (no switch), (2) switched between sentences, with a different talker masker (new switch), or (3) switched between sentences, and included the target talker of the first sentence as a masker (old switch). The two nonspeech masking conditions included no switch and new switch manipulations.

Talkers and callsigns were counterbalanced across target and masker stimuli within each condition. colors and number were different for each sentence within a trial (e.g., if sentence 1 was “Blue-One,” neither “Blue” nor “One” could occur in target sentence 2 or the masker). Each color–number combination was also only presented once for each sentence in a condition (e.g., in the no switch noise condition “Blue-One” only occurred in sentence 1 once). In total, participants completed 140 randomized trials (28 per condition).

### Procedure

Participants took part in two online surveys separated on average by seven days. Session one took on average of 25 min to complete and session two took on average 50 min. At the beginning of each survey participants were instructed to remove hearing aids if applicable and use headphones for the duration of the study. Prior to beginning the listening tasks (i.e., the DTT and talker switching task) participants completed a headphone adjustment task (using steady-state speech noise prior to the DTT and CRM sentences prior to the CRM task) to ensure the stimuli were at a comfortable listening level.

In session one each participant completed screening consisting of the hearing questionnaires and the DTT. In the older adult group, scores on the HHIE and SSQ, as well as self-reported hearing ability, were considered in combination to distinguish a cluster of participants forming a “poorer perceived hearing” group, and a cluster of participants forming a “better perceived hearing” group, for invitation to session 2. The better hearing group included those who responded “No” to a perceived hearing loss question (“Do you feel you have a hearing loss?”), and who scored ≤ 16 on the HHIE screener (indicating no handicap). The poorer hearing group included those who responded “Yes” to a perceived hearing loss question, and who scored > 16 on the HHIE screener (indicating hearing handicap).

In addition, to further ensure distinction between groups (given the lack of control available when conducting studies online, and propensity for individual error), we required ratings from the SSQ to be consistent with their grouping (i.e., low SSQ scores for the better hearers, and high SSQ scores for the poorer hearers). We therefore used the sample's median score of 7 as a threshold and removed any participants whose SSQ score conflicted with their grouping based on the perceived hearing loss question and HHIE screener. Of the participants included in the final analysis, all 22 young adults fell into the “better perceived hearing” group, 22 of the older adults fell into the “better perceived hearing” group, and 21 older adults fell into the “poorer perceived hearing” group.

Session two included the speech following task. They were instructed to remember the color-number combination produced in the first and second target sentences (assigned with the same callsign) while ignoring the masker. After hearing both sentences two grids appeared on the screen. Participants responded by selecting the corresponding color–number combination in the grid for both the first and second target sentences. Demonstration and practice trials were provided to ensure that participants understood the task.

### Data Analysis

The number of correct responses was calculated for sentence 1 and sentence 2 separately. For sentence 2 in the speech masking trials only, we also calculated the number of masker confusions compared to other errors. Masker confusions were incorrect selections of target words spoken by the masker. Other errors were incorrect selections of colors or numbers not spoken in either sentence 2 or the masking sentence. Scoring was out of two key words per sentence (color and number), and thus each condition was summed out of a possible 56 (2 words per sentence * 28 trials). A total of 5 participants with accuracy scores more than 2.5 *SD* from the full sample mean in more than one condition were excluded (all showing poorer performance either in sentence 1 or sentence 2).

Data were analyzed using Bayesian regression using the R-INLA package (Martins et al., 2013; Rue et al., 2017). Models were fitted for each different response outcome (i.e., sentence 1 accuracy, sentence 2 accuracy, masker confusions, and other errors) for noise and speech masked trials. As we were primarily interested in the effects of switching attention and hearing ability, the main effects of switch condition and SRT were included in all models. Multiple candidate models were fitted for each response outcome in order to assess the predictive performance of including fixed effects of age group, SSQ score, as well as interactions between each main effect term with switch condition. Models that best fit the data were selected based on Widely Applicable Information Criteria (WAIC; Gelman et al., 2014) measures. In addition, all final models contained a random intercept for each participant to account for dependencies stemming from repeated measures.

Odds-ratios were calculated for each of the main effects, to measure the strength of association between the factor of interest (i.e., switch condition or hearing measures) and the accuracy of response. Odds ratios for switch conditions represent the change in likelihood of an outcome for each condition compared to the no switch condition. Scores for hearing measures were standardized to have a mean of zero and standard deviation of one. Odds ratios for hearing measures represent the change in likelihood of an outcome for a 1 standard deviation (*SD*) increase in each predictor. This standardization allows for a more interpretable comparison across variables on a common scale. A 95% credible interval for each odds ratio was computed to assess statistical significance. The statistical significance of an odds ratio at the 95% level can be inferred if the credible interval does not include 1 (Wang et al., 2018).

## Results

### Demographics

For simplicity, demographics are reported using participants’ self-reported hearing ability as a grouping factor (see Table 2). However, for the statistical analyses below, we instead used the more objective SRT (DTT) as a measure of hearing ability.

**Table 2.** Demographic Information and Scores on Hearing Measures (Means and Standard Deviations).

	All	Young	Old – better perceived hearing	Old – poorer perceived hearing
Age		26.38(2.96)	70.18(4.68)	70.62(3.77)
SRT (DTT)	−16.74(5.11)	−20.85(2.44)	−16.19(4.19)	−13.01(5.03)
SSQ-12	6.99(1.92)	7.42(1.23)	8.48(0.75)	4.98(1.64)
HHIE	14.31(19.49)	1.91(2.93)	2.91(3.79)	39.29(15.05)

### Noise Masking

Noise models were fitted with main effect terms switch condition, SRT and SSQ score only, as model fit was not improved by inclusion of terms for age group, or an interaction between switch condition and speech-reception threshold. Note that SSQ score improved model fit for sentence 2 accuracy only. Odds ratios for the effects on accuracy are reported in Table 3.

**Table 3.** Modulated Noise Masker: Odds Ratios for Effects of Switch Condition, SRT (DTT), and SSQ Score on Sentence 1 and Sentence 2 Accuracy With 95% Credible Intervals.

Modulated noise masker		
	Sentence 1 accuracy	Sentence 2 accuracy
Predictors	Odds ratios [95% CI]	
New switch	1.36 [0.93, 1.99]	1.21 [1.00, 1.47]
SRT (DTT)	0.87 [0.62, 1.23]	0.62 [0.53, 0.72]*
SSQ score	0.99 [0.70, 1.38]	1.18 [1.03, 1.37]*

*Note*. SRT and SSQ scores are standardized scores. Estimates reflect 1 *SD* of the mean.

*Significant effects.

#### Accuracy

For sentence 1, none of our predictors had significant effects on response accuracy, potentially as participants performed close to the ceiling in both No Switch (*M* = 98.27%, *SD* = 2.42%) and New Switch (*M* = 98.71%, *SD* = 1.98%) conditions.

For sentence 2, we saw no significant effect of the switch condition. However, we did see a negative effect of SRT, whereby participants requiring higher signal-to-noise ratios to receive digits in noise were less accurate than those requiring lower SRTs. Specifically, for each 1 *SD* increase in SRT, a listener's odds of selecting the correct target words were reduced by 38%. There was also a complementary positive effect of SSQ, whereby participants with higher self-rated hearing abilities were more accurate than participants with lower self-rated hearing abilities, reflecting an 18% increase in the odds of selecting the correct target words for each 1 *SD* increase in SSQ. Note sentence 2 also had high accuracy in both no switch (*M* = 92.94%, *SD* = 5.81%) and new switch (*M* = 94.07%, *SD* = 5.93%) conditions.

#### Errors

Note that in this condition, errors would be the inverse of accuracy and therefore are not reported.

### Speech Masking

Speech models were fitted with main effect terms switch condition, SRT, and an interaction between switch condition and SRT only, as we did not find effects of age group or SSQ score to improve model fit. Note that interaction between switch condition and SRT improved model fit for sentence 2 accuracy and masker confusions only. Odds ratios for the effects on accuracy are reported in Table 4 and the effects on errors are reported in Table 5.

**Table 4.** Speech Maskers: Odds Ratios for the Effects of Switch Condition, Speech-Reception Threshold and an Interaction Between Switch and Speech-Reception Threshold on Sentence 1 and Sentence 2 Accuracy With 95% Credible Intervals.

Speech maskers		
	Sentence 1 accuracy	Sentence 2 accuracy
Predictors	Odds ratios [95% CI]	
New Switch	0.88 [0.71, 1.09]	0.71 [0.64, 0.79]*
Old Switch	0.77 [0.62, 0.95]*	0.45 [0.41, 0.50]*
SRT (DTT)	1.05 [0.77, 1.43]	0.77 [0.68, 0.88]*
New Switch x SRT (DTT)	0.89 [0.72, 1.11]	1.12 [1.01, 1.24]*
Old Switch x SRT (DTT)	1.06 [0.86, 1.32]	1.06 [0.96, 1.17]

*Note*. SRT is a standardized score. Estimates reflect 1 *SD* of the mean.

*Significant effects.

**Table 5.** Speech Maskers: Odds Ratios for the Effects of Switch, Speech-Reception Threshold and Interaction Between Switch and Speech-Reception Threshold on Masker Confusions, and Other Errors With 95% Credible Intervals.

Speech maskers		
	Masker confusions	Other errors
Predictors	Odds ratios [95% CI]	
New Switch	1.42 [1.28, 1.59] *	1.03 [0.78, 1.35]
Old Switch	2.18 [1.97, 2.42] *	1.38 [1.07, 1.79] *
SRT (DTT)	1.29 [1.14, 1.46] *	1.17 [0.92, 1.50]
New Switch × SRT (DTT)	0.86 [0.77, 0.95] *	1.24 [0.95, 1.61]
Old Switch × SRT (DTT)	0.89 [0.80, 0.99] *	1.28 [1.00, 1.64]

*Note*. SRT is a standardized score. Estimates reflect 1 *SD* of the mean.

*Significant effects.

#### Accuracy

**Figure 3.**
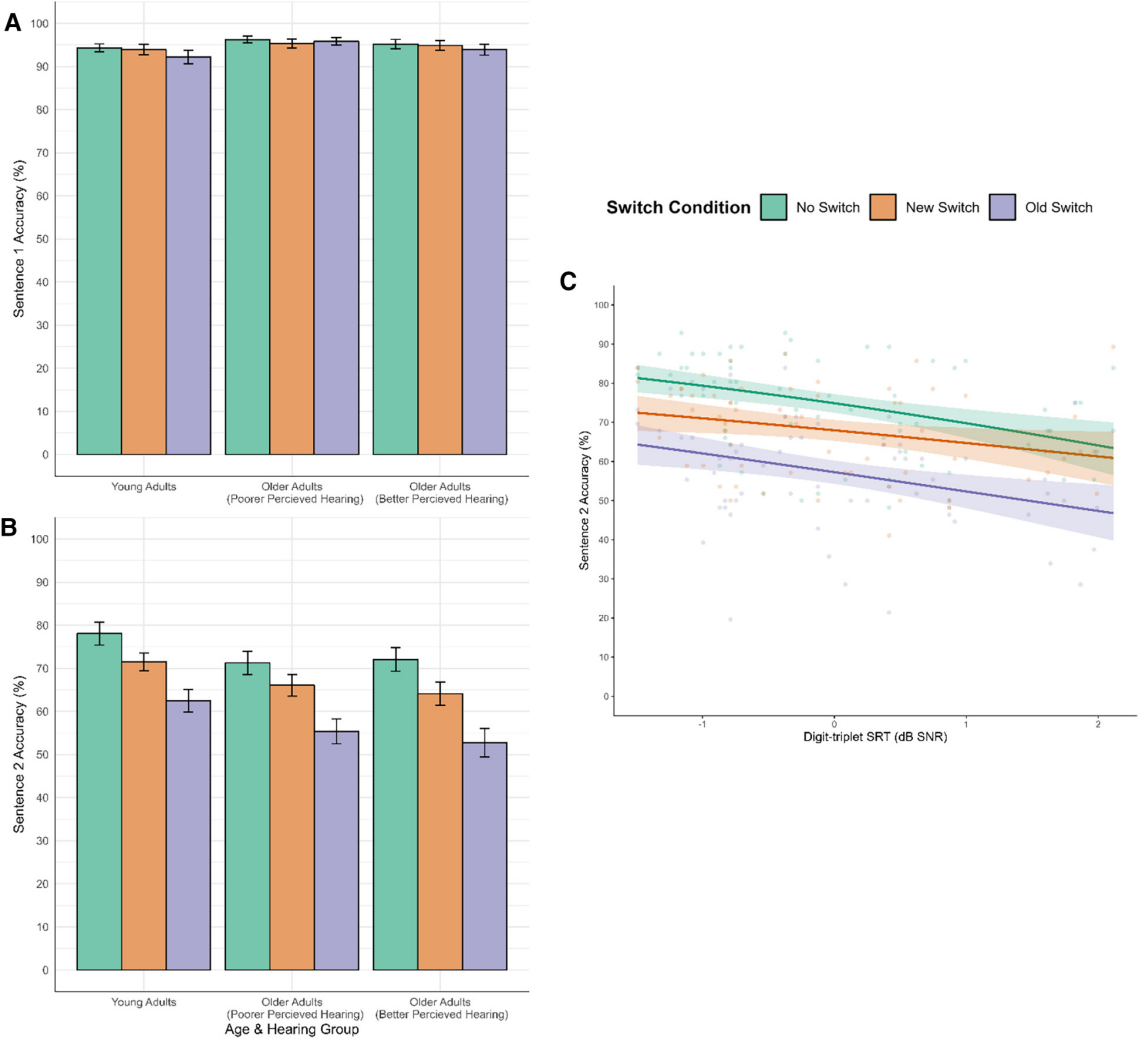
(A) Average sentence 2 masker confusions, and (B) observed data points and model estimates for the effect of SRT (DTT) on sentence 2 masker confusions. Regression lines are plotted with 95% confidence intervals.

For sentence 1, whilst there was no significant difference in accuracy between the no switch (*M* = 95.25%, *SD* = 4.41%) and new switch (*M* = 94.70%, *SD* = 5.33%) conditions (see Table 4, Figure 3A), there was a significant (although very small) effect of old switch condition (*M* = 93.96%, *SD* = 6.12%), corresponding to a 23% reduction in odds of selecting the target word in the old switch compared to the no switch condition. There was also no significant difference between the old switch and new switch condition; 0.87 [0.71, 1.07]. No other predictors were found to have a significant effect on sentence 1 accuracy.

**Figure 4.**
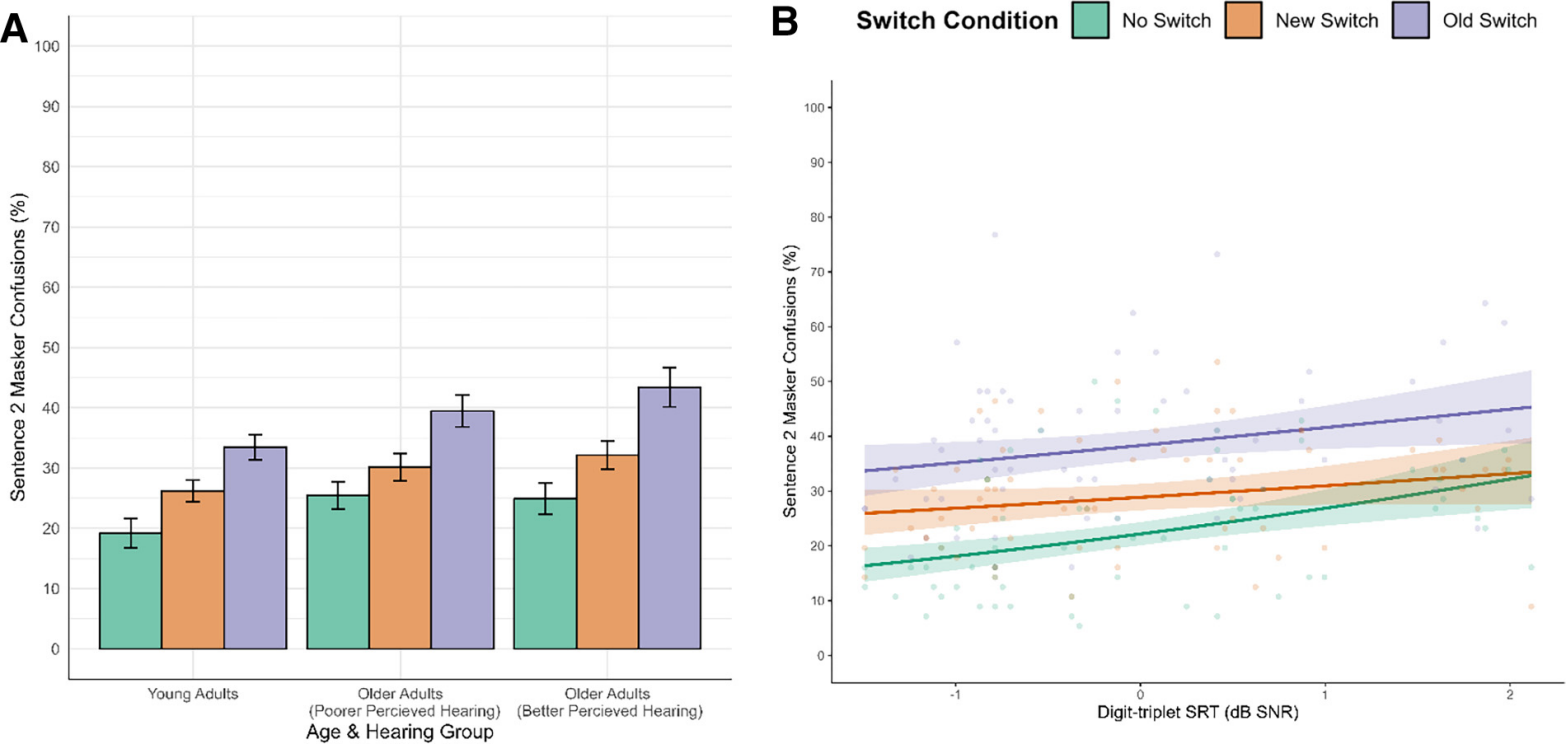
(A) Average sentence 1 accuracy, (B) average sentence 2 accuracy, and (C) observed data points and model estimates for the effect of SRT (DTT) on sentence 2 accuracy. Regression lines are plotted with 95% confidence intervals.

For sentence 2, accuracy was associated with a number of factors. There was a significant effect of switch condition on sentence 2 accuracy, with accuracy being higher in the no switch (*M* = 73.85%, *SD* = 12.71%) condition than either New (*M* = 67.25%, *SD* = 11.50%) or Old (*M* = 56.90%, *SD* = 14.22%) switching conditions (see Table 4, Figure 3B). Specifically, the new switch condition was associated with a 29% reduction in the odds of a correct response, while the old switch condition showed a larger 55% reduction in the odds of a correct response. Furthermore, accuracy was particularly low when the switch required disengaging from the previous target talker, with accuracy being lower in the old switch than new switch conditions (old versus new switch; 0.63 [0.57, 0.70]). There was also a negative relationship between SRT and sentence 2 accuracy; higher thresholds were associated with lower accuracy, corresponding to a 23% reduction in odds of selecting sentence 2 target words for each 1 *SD* increase in SRT. This effect was modulated by switch condition (see Figure 3C). Specifically, the effect of SRT was reduced in the new switch condition relative to the no switch condition. In contrast, the interaction between SRT and old switch did not reach significance, indicating the effect of SRT is similar in the old and no switch conditions.

#### Errors

In terms of masker confusions, there was a significant effect of switch condition (see Table 5, Figure 4A). The odds of a masker confusion were 42% higher in the new switch condition (*M* = 29.51%, *SD* = 10.14%) and 118% higher in the old switch condition (*M* = 38.76%, *SD* = 13.16%) versus when the talker remained the same (*M* = 23.13%, *SD* = 11.46%). Participants performed particularly poorly when required to disengage from the old target talker than just follow a new talker (old versus new switch; 1.53 [1.39, 1.69]). There was also a significant positive effect of SRT on masker confusions; for each 1 *SD* increase in SRT there was a 29% increase in odds of producing a masker confusion. However, the relationship between SRT and masker confusions was modulated by switch condition (see Figure 4B), such that the effect was not evident in the new switch condition, and stronger in the no switch relative to the old switch condition.

Finally, while other errors were low, participants made significantly more in the old switch (*M* = 4.34%, *SD* = 3.77%) than either the no switch (*M* = 3.02%, *SD* = 3.28%) condition, or the new switch (*M* = 3.24%, *SD* = 3.28%) condition; 1.35 [1.04, 1.75]. No other predictors were found to significantly affect other errors.

## Discussion

This study examined the process of switching nonspatial attention in multitalker conversation with either noise or a competing talker. Lin and Carlile (2019) previously examined the effects of switching nonspatial attention in young adults with normal hearing and found worse performance when listeners were required to follow a switch in target talker. While subsequent work has indicated that this is likely to also occur in older adults (Meister et al., 2020), there are indications that hearing loss may impair performance, particularly when the talker remains constant (Wächtler et al., 2022). In this study we extended this work to explore the effects of age and hearing using a continuous measure of ability, analyzing ability to follow turn switches in terms of both speech recall accuracy and types of errors produced. Critically, we examined the recall of sentences occurring before and after a turn-taking gap.

While we saw no effect of switch type in a noise masker (likely due to participants’ high accuracy level), we replicated prior effects in the speech masking conditions, whereby listeners showed greatest recall when target sentences were produced by the same talker, reduced recall when the target talker changed, and worst recall when a new target talker occurred in the presence of an old target talker masker. Indeed, an exploratory individual-level analysis confirmed that the majority of participants followed this pattern of performance (for 36 of the 65 participants, the ordering of the three speech masked conditions was No Switch > New Switch > Old Switch. When looking only between pairs of conditions, a total of 46 scored better for No Switch than New Switch, and a total of 54 scored better for New Switch than Old Switch). Strikingly, across both noise and speech masking conditions we saw no benefit of including age group in our models but did see significant effects of hearing ability (as assessed via the DTT). Of particular note is the specificity of the effect of hearing ability: in the speech masking conditions an interaction between switch type and hearing ability showed that the effect of hearing ability was stronger in the no switch condition than the new switch condition. Differences in SRT affected accuracy and masker confusions to a greater extent in the no switch condition than in the new switch condition, with Figures 3 and 4 suggesting that this was due to less of a benefit of the talker remaining the same for individuals with poorer hearing.

### Effect of Switching

In detail, although we saw no effect of switch condition on accuracy in the noise masking trials, all listeners performed at a high level with average correct responses exceeding 93% for both sentences. Nonetheless, in the more challenging speech masking trials, we did see an effect of switch condition, with participants being less accurate in selecting sentence 2 words when there was a change in talker after the gap. Lin and Carlile (2019) attributed these *listening costs* to the cognitive demands of reengaging and disengaging attention. They reported that speech recall accuracy was best when the talker remained the same across target sentences, a condition in which listeners can use a priori information about the characteristics of the target talker's voice to guide attention (Kidd et al., 2005). However, when the target talker changes, cognitive demands are greater, as listeners have to identify and reorient attention to a new target. The decrease in accuracy and increase in masker confusions that we find for the switching conditions likely reflects this cost of reengaging attention.

When the target talker becomes the masker, cognitive load is further increased, since listeners must also disengage from (and inhibit) the previous target. Listeners may develop a perceptual bias towards a previously attended target talker which can hinder performance when required to switch attention to a new talker (Best et al., 2008). In support of this, Lin and Carlile (2015) found that young normal hearing listeners experienced a decrease in word recall and increase in masker confusions when the target talker became the masker (old switch condition). We replicated this finding in the current study, with the old switch condition leading to poorer sentence 2 accuracy, increased masker confusions, and more other errors than the new switch condition across all participants. Together these findings suggest a failure to disengage attention from the prior target talker, and reorient attention to the correct talker. This was also the only condition to significantly differ from the same talker condition (though this was a very small difference) in terms of the accuracy of recalling sentence 1 (presented in quiet). As recall of both sentences occurred after the second had occurred, this impact on recall of the easily audible earlier sentence indicates the challenge that this condition posed for participants. Our results therefore provide further evidence of the costs associated with switching attention between talkers, and extend this to older adults with a range of hearing abilities.

### Effect of Hearing Ability

In both speech and noise trials, poorer SRTs were associated with reduced ability to follow the utterances after a turn taking gap. Participants with higher speech reception thresholds (i.e., poorer hearing) were less accurate at selecting target coordinates in the second sentence in both noise and speech masked trials, and also made more errors. Moreover, for noise masked trials a complimentary effect of SSQ score was found to be associated with sentence 2 accuracy, whereby participants with better self-rated hearing were more accurate at recalling target words than participants with lower scores. (Note that we did not however see a relationship between SSQ in the speech masked trials, potentially due to SSQ variability).

Overall reduced accuracy for listeners with higher SRTs (i.e., poorer hearing) might be partially attributed to decreased audibility of the target signal. This is consistent with findings from Wächtler et al. (2022), who found that older hearing-impaired listeners showed a higher rate of errors compared to groups of young normal hearing listeners. However, in our speech masked trials, the majority of errors were confusions (30.47%), with the rate of other errors being very low (<4%), and not being associated with SRTs. If the decline in performance associated with increased SRTs was simply due to decreased audibility then we could have expected other errors to have made up the majority of errors made. Instead, the vast majority of incorrect responses to sentence 2 were confusions with the masking sentence. As such, it appears that stimuli were audible, but that worse performance for sentence 2 with hearing loss was driven by difficulty separating and identifying the target from the masking signal.

Notably, the effect of SRTs in speech masked trials appeared to be modulated by switch condition. Overall, higher SRTs were associated with worse performance (i.e., decreased sentence two accuracy, and increased masker confusions). However, the effect of SRTs was stronger when the target voice remained the same (no switch) than when it switched to a new talker (new switch), for both sentence 2 accuracy and masker confusions.

In further detail, for participants with better hearing in noise there was a larger difference in accuracy between no switch and new switch conditions, with higher accuracy in the no switch condition than the new switch condition. This suggests that the listeners with better hearing showed a benefit from the talker remaining the same. However, for participants with poorer hearing in noise, the difference between these conditions was reduced. This suggests that the listeners with poorer hearing specifically had more difficulty utilizing consistent talker characteristics to focus their attention. Previous research shows that listeners with normal hearing can use a priori voice-specific information (such as fundamental frequency and VTL cues; Darwin et al., 2003) to follow the voice of a particular target talker and ignore irrelevant talkers. However, listeners with poorer hearing have been shown to be less able to use such information for stream segregation (Mackersie et al., 2011; Summers & Leek, 1998) which could contribute to their poorer performance in the same voice condition specifically. Consistent with our findings, Wächtler et al. (2022), reported that while listeners with poorer hearing showed significantly more errors than listeners with normal hearing during trials in which the target talker remained the same, there was no difference between groups when the target talker switched. Thus, this may suggest that poorer hearing reduces the benefit gained from a voice remaining consistent between utterances, potentially due to poorer object formation or streaming for the original target voice. Interestingly we acknowledge that we did also see an interaction between SRT and old switch masker confusions, with listeners with poorer hearing still having more difficulty disengaging from the original target than when listening in the presence of a new talker masker. While surprising, this could suggest that benefitting from a consistent talker and inhibiting a previously heard talker, involve different cognitive processes.

### Limitations

This study involved an online assessment of speech listening and thus has a number of limitations. Since data was collected online, we were not able to collect clinical audiometric data from participants, instead using SRT (DTT score) as an approximation of hearing ability. While of course SRT may have been affected by factors such as presentation level or quality (dependent on a participant's personal equipment), we chose this metric (e.g., as opposed to the SSQ) since any equipment-induced noise would presumably also present in the main turn-following task, and thus remove some variability from our data. Despite differences in assessment methods, average sentence 2 accuracy for young adults with “better perceived hearing” in our study (No Switch = 78.08%, New Switch = 71.51%, Old Switch = 62.50%) was comparable to the data obtained by Lin and Carlile (2019) for young adults with normal hearing, as assessed by a pure tone-audiogram (No Switch = 85.6%, New Switch = 76.7%, Old Switch = 75.4%), with participants in both studies showing similar patterns of performance. Thus, performance levels in the online task were consistent with those observed in a more controlled lab-based setting.

It is also possible that some variance in the talker switching conditions is related to the complexity of the task itself. Indeed, an exploratory analysis showed that participants were more accurate in recalling sentence two target words and made less masker confusions in the speech conditions in which the target began before the masker, suggesting an effect of attention on task performance. Additionally, in the condition where the talker remains the same the listener simply has to continue the same task of understanding that talker. However, in the talker switching conditions, participants have an additional task of reorientating their attention to the target talker. It is also possible that participants may experience some confusion about the task when the target talker becomes the masker, which may momentarily affect their attention, resulting in worse performance. However, as the majority of participants’ responses were indeed selections of the correct target words (even in the switching conditions), it appears participants were able to understand and complete the task.

Another limitation of this study is that although the paradigm included many components of conversational turn taking, it nonetheless remains removed from a real conversation situation due to its artificiality. Critically, simultaneous presentation of target and masker in sentence 2, in conjunction with uncertainty as to the identity of the target (due to the callsign being uttered as the second word) is unlike typical cocktail party listening. Furthermore, participants’ engagement with the speech will have been quite different to in a real conversation, both since they did not need to listen with the goal of responding and furthering the conversation, and because the sentence stimuli were not typical “turns,” in that they did not combine to build upon a topic. Future work using more naturalistic conversational sentence stimuli, that include alternative forms of context to signal talker changes, may reveal compensation strategies that listeners may make use of to ameliorate difficulty in such tasks.

## Conclusions

This study addressed the effect of aging and hearing ability on recall of sentences spoken by the same or different talkers. We replicated prior findings that listeners are better able to follow sequential sentences spoken by the same talker than different talkers. We also saw that listeners with poorer hearing had poorer recall of the second target sentence (whether it was masked by noise or speech), suggesting that hearing affects a listener's ability to use information from one sentence (i.e., a callsign) to accurately identify and follow a second sentence. Finally, we found that listeners with poorer hearing were particularly impacted when the talker remained the same between sentences, suggesting that they did not show the same “consistent talker benefit” seen in the better hearing participants. This work sheds light on additional difficulties around turn following that could be driven by hearing difficulties, suggesting that cognitive processes involved in engaging and disengaging attention to specific talkers deserve further investigation.
